# Exploration of genotype-by-environment interactions affecting gene expression responses in porcine immune cells

**DOI:** 10.3389/fgene.2023.1157267

**Published:** 2023-03-16

**Authors:** Eduard Murani, Frieder Hadlich

**Affiliations:** Institute of Genome Biology, Research Institute for Farm Animal Biology (FBN), Dummerstorf, Germany

**Keywords:** genotype-by-environment interaction, G×E, allele-specific expression (ASE), eQTL, cis-regulatory variation, pig, PBMC

## Abstract

As one of the keys to healthy performance, robustness of farm animals is gaining importance, and with this comes increasing interest in genetic dissection of genotype-by-environment interactions (G×E). Changes in gene expression are among the most sensitive responses conveying adaptation to environmental stimuli. Environmentally responsive regulatory variation thus likely plays a central role in G×E. In the present study, we set out to detect action of environmentally responsive *cis*-regulatory variation by the analysis of condition-dependent allele specific expression (cd-ASE) in porcine immune cells. For this, we harnessed mRNA-sequencing data of peripheral blood mononuclear cells (PBMCs) stimulated *in vitro* with lipopolysaccharide, dexamethasone, or their combination. These treatments mimic common challenges such as bacterial infection or stress, and induce vast transcriptome changes. About two thirds of the examined loci showed significant ASE in at least one treatment, and out of those about ten percent exhibited cd-ASE. Most of the ASE variants were not yet reported in the PigGTEx Atlas. Genes showing cd-ASE were enriched in cytokine signaling in immune system and include several key candidates for animal health. In contrast, genes showing no ASE featured cell-cycle related functions. We confirmed LPS-dependent ASE for one of the top candidates, *SOD2*, which ranks among the major response genes in LPS-stimulated monocytes. The results of the present study demonstrate the potential of *in vitro* cell models coupled with cd-ASE analysis for the investigation of G×E in farm animals. The identified loci may benefit efforts to unravel the genetic basis of robustness and improvement of health and welfare in pigs.

## 1 Introduction

Characterization of the genetic variation in human (Lappalainen et al., 2019) and livestock populations (Hayes and Daetwyler, 2019) showed that each individual carries several thousand functional variants, majority of them non-coding (Auton et al., 2015; GTEx Consortium, 2020). These do not act in isolation, but in the context of the genetic background and environment of the carrier. To unravel mechanisms behind the genotype-phenotype connection we thus have to tackle the next challenge and explore interactions between genetic variants with the genetic background and environment as well. Disease risk, drug response, and stress resilience are some prominent examples of traits in humans and farm animals where genotype-by-environment interactions (G×E) play a considerable role (Rauw and Gomez-Raya, 2015; Elbau et al., 2019). Severe environmental consequences of the climate change, particularly extreme temperatures and the resulting physiological challenges to cope with them (Koch et al., 2019), sparked increased interest in recent years in research on G×E in farm animals to increase their resilience (Passamonti et al., 2021). Currently, investigation of G×E in farm animals relies largely on quantitative genetic and QTL mapping approaches to measure G×E and to identify the underlying loci (e.g., Freitas et al., 2021). Such studies generally require large cohorts with phenotypes recorded in different environments.

Functional genomics offers complementary, yet in farm animals so far rarely applied, approaches to explore G×E and the underlying biological and genetic mechanisms (Ritchie et al., 2017). Gene expression can be regulated in three dimensions—transcript abundance, time (e.g., ontogeny, or dynamics) and space (cell type)—and thus allows rapid, fine-tuned response and adaptation to environmental stimuli (López-Maury et al., 2008). Accordingly, genomic loci influencing gene expression (expression QTL—eQTL) were evidenced as important drivers of adaptation (Quiver and Lachance, 2022). Current omics technologies allow genome-wide, holistic analysis of gene expression which thus represents a molecular endophenotype well suited to study G×E. Many studies exploring G×E in gene expression leveraged *in vitro* cellular systems that enable testing of a wide variety of stimuli and better control of the environmental conditions (Moyerbrailean et al., 2016; Findley et al., 2021; Lea et al., 2022). For the identification of environmentally responsive eQTL (also referred as reQTL), and thus G×E, two approaches were devised. The first approach employs QTL-mapping such as genome-wide association study (GWAS) (Fairfax et al., 2014; Kim et al., 2014), and requires relatively large sample sizes. Alternatively, reQTL can be detected by analyzing allele-specific expression (ASE) (Edsgärd et al., 2016; Moyerbrailean et al., 2016; Knowles et al., 2017). Mapping of ASE loci is a powerful approach to detect genes affected by *cis*-eQTL, i.e., by genetic variation influencing function of the cognate regulatory elements (Castel et al., 2015). We previously applied this method for the dissection of the complex genetic background of tissue-specific *cis*-regulatory variation of a single locus (Murani et al., 2013). Because ASE is measured using allelic ratios of transcribed SNPs within a sample, and thus matched background, this reduces noise and consequently the required sample size. On the other hand, the transcribed SNPs (here termed cSNPs) can be called directly from RNA-seq data, essentially obviating the need for genotyping to detect ASE. To investigate G×E, the ASE approach has been extended by comparing ASE between different conditions (designated condition-dependent ASE, cd-ASE) using a variety of statistical models (Mayba et al., 2014; Edsgärd et al., 2016; Moyerbrailean et al., 2016; Knowles et al., 2017; Fan et al., 2020). Importantly, loci evidenced to be influenced by G×E using ASE analysis were enriched in GWAS signals (Moyerbrailean et al., 2016), indicating that they contribute not only to expression divergence, but to phenotypic variation as well.

In the present study, we set out to identify genes influenced by environmentally responsive *cis*-regulatory variation in porcine immune cells using ASE analysis. To this end, we harnessed mRNA-Seq data from porcine peripheral blood mononuclear cells (PBMC) stimulated *in vitro* with either endotoxin (LPS), dexamethasone (DEX), or their combination (Li et al., 2021). These treatments model inflammation, neuroendocrine stress response, and neuroimmunomodulation, respectively. We performed SNP- as well as gene-wise analyses and identified a number of genes showing evidence of G×E in their response to treatment. These genes represent candidates for the improvement of health and welfare in pigs.

## 2 Materials and methods

### 2.1 *In vitro* stimulation of PBMC, RNA extraction, and cDNA synthesis

The initial cell experiment has been described in detail by Li et al. (2021). Briefly, PBMC isolated from blood collected during exsanguination at slaughter of 24 individuals (balanced for sex) were stimulated for 2 h with either vehicle (C), 5 nM of DEX (D), 10 μg/mL of LPS (L), or LPS (10 μg/mL) + DEX (5 nM) (LD), respectively. For the independent verification of cd-ASE of *SOD2*, informative PBMC samples were selected based on genotype of the marker MARC0005058 (rs80857128) obtained from 60 K Illumina SNP array data published previously (Reyer et al., 2019). As before, the PBMCs were obtained from slaughter blood and cryopreserved until use. The genotype was validated using Sanger Sequencing as described below. The cell experiment using the new set of PBMCs was repeated essentially as described previously (Li et al., 2021), but only vehicle and 10 μg/mL of LPS were applied in the validation experiment. After 2 h stimulation, cells were harvested for RNA extraction. The cell pellet was lysed in TRI-Reagent (Sigma-Aldrich, Taufkirchen, Germany). After phase separation, total RNA was isolated using the Direct-zol RNA Miniprep (Zymo Research, Freiburg, Germany), including on column DNase-digestion. cDNA was synthesized using SuperScript III MMLV reverse transcriptase (Invitrogen, Karlsruhe, Germany) in a reaction containing a mixture of 500 ng random hexamers (Promega, Mannheim, Germany), 500 ng of oligo (dT)11 VN primer, 40 U of RNAsin Plus (Promega, Mannheim, Germany) and 500 ng total RNA.

### 2.2 cSNP calling, read counting, and haplotype analysis of candidate genes

The mRNA-Sequencing experiment has been performed previously at the Institute of Genome Biology (FBN) on an Illumina HiSeq 2500 sequencing platform. In total 96 transcriptome profiles were generated *via* 2 × 101 bp paired-end sequencing. The resulting average sequencing depth was >20 M read pairs with an alignment rate of ∼98% (Li et al., 2021). The sequencing data were deposited at the ArrayExpress repository under the accession number E-MTAB-9808.

SNP calling and counting of reads per allele was essentially based on the pipeline described by Tomlinson et al. (2021). In more detail, the mRNA-Seq reads were aligned to Sus.Scrofa 11.1 reference genome (release 103) using STAR (v.2.7.8a) (Dobin et al., 2013) in the 2-pass mode, and filtered to remove duplicates and reads with ambiguous mapping using samtools (version 1.12) (Danecek et al., 2021). After that, aligned reads were processed *via* the Genome Analysis Toolkit (GATK) pipeline (version 4.2.0.0) (DePristo et al., 2011) including splitting reads by cigar string to detect splicing events, base recalibration, and variant calling using HaplotypeCaller. For variant calling the phred-scaled confidence threshold was set to 20, and soft-clipped bases were avoided. The gVCF files thus obtained from the individual mRNA-seq profiles were then aggregated using bcftools (1.10.2) (Danecek et al., 2021), and the resulting collection of discovered variants was used to N-mask the reference genome using bedtools (version v2.27.1) (Quinlan and Hall, 2010). The process of read mapping and variant calling was subsequently repeated using the N-masked genome in order to remove potential allelic mapping bias (Castel et al., 2015), but in the second run, the pipeline included also removal of biased reads using WASP (van de Geijn et al., 2015).

To analyze haplotype structure of *SOD2*, the genotypes of cSNPs residing within the transcriptional unit were retrieved from the compiled global VCF file. Haplotype structure was first explored in Haploview 3.2 (Barrett et al., 2005). Afterwards the individual diplotypes were determined using the EM method implemented in JMP Genomics v10.1 (SAS Institute, Cary, NC, United States).

### 2.3 ASE analysis using mRNA-Seq data

In addition to quality control measures during the previous steps, SNPs entering the ASE analysis were filtered based on their read coverage as suggested by Castel et al. (2015). Thus only biallelic sites covered in heterozygous state by at least 30 reads in total, having at least three reads per either allele, and a contribution per allele of at least 1% to the total number of reads, were retained for further analyses. SNPs located on sex chromosomes and unmapped SNPs were omitted.

For SNP-wise ASE analysis within condition, the VCF ASE Detection Tool (VADT) software (Tomlinson et al., 2021) was employed. To test ASE for informative variants passing the filtering steps, the VADT software performs a binomial test. The *p*-values of individual samples were subsequently combined per SNP across the tested heterozygous samples using Fisher’s method, and finally adjusted across all tested informative SNPs using the Benjamini–Hochberg procedure to control the false discovery rate (FDR). In order to perform SNP-wise cd-ASE analysis we applied Fisher’s exact test as suggested by Edsgärd et al. (2016). For this, sample-wise 2 × 2 contingency tables of read counts were calculated for SNPs passing above-mentioned filtering criteria in each of the analyzed pair of conditions in at least one heterozygous individual. The individual *p*-values were combined and FDR-adjusted as described for the static ASE analysis above.

For gene-wise ASE analysis the GeneiASE software (Edsgärd et al., 2016) was applied. Similar to the SNP-wise cd-ASE analysis, GeneiASE calculates contingency tables for allele counts of informative SNPs (either 2 × 1 or 2 × 2 table for the analysis within or between conditions, respectively). Based on this, a test statistic is calculated for genes covered by at least two SNPs, combining the SNP-wise effects *via* meta-analysis. A *p*-value was computed on the basis of a null distribution derived by resampling (*n* = 10,000), and then combined and adjusted as described for the SNP-wise analysis.

Haplotype-level allelic ratios of *SOD2* were calculated based on allele counts of the 21 SNPs included in the ASE analysis, and the phase information obtained from haplotype analysis. Haplotype-level allelic ratios were compared using one-way ANOVA with Dunnett test implemented in GraphPad Prism 9.4.1 (GraphPad Software, San Diego, United States). Fold changes of *SOD2* expression due to treatment were calculated based on normalized count data from our previous study and log2 transformed (Li et al., 2021). The effect of diplotype on the individual fold-changes was examined using one-way ANOVA with Šidák test implemented in GraphPad Prism 9.4.1.

### 2.4 Locus-specific ASE analysis

A 600bp-long PCR-fragment of 3′ UTR of *SOD2* was designed (forward primer: CGT​CAG​ACC​TGA​TTA​CCT​GAA​AGC; reverse primer: CTA​AAG​ACC​ACT​GGG​TGG​TAC​CTG) using the web-based tool Primer3. The PCR fragment covered three *SOD2* cSNPs exhibiting cd-ASE: rs80848798, rs80857128, and rs80857128. The PCR fragment was amplified in a 20 µL reaction mixture containing 2 µL cDNA, 0.2 µM of each primer, 50 µM of each dNTP and 0.5 U SupraTherm Taq Polymerase (Ares Biosciences, Cologne, Germany) in ×1 supplied PCR-buffer containing 1.5 mM MgCl2. The temperature profile was as follows: denaturation at 95°C for 15 s, annealing at 60°C for 60 s and extension at 72°C for 60 s for 40 cycles. The PCR products were checked on 2% agarose gels and cleaned-up using magnetic beads (Illumina, San Diego, United States). Sanger sequencing was performed by the commercial provider Genewiz (Azenta, Leipzig, Germany) using the forward primer. The resulting sequence chromatograms were analyzed using the PeakPicker V0.5 software (Ge et al., 2005) essentially as described previously (Murani et al., 2013). The normalized peak ratios were compared between the C and L treatments using a paired *t*-test in GraphPad Prism 9.4.1.

### 2.5 Analysis of functional enrichment and upstream regulators of ASE genes

To reveal which functional pathways are enriched among genes exhibiting different ASE properties (see below in the results section) a list comprising all five groups of genes (cd-ASE in three different conditions, static ASE, and no ASE) was analyzed using the pick selective GO clusters option of the web-based gene annotation tool Metascape (Zhou et al., 2019). Potential upstream regulators were predicted from individual gene lists using Ingenuity Pathway Analysis (QIAGEN, Hilden, Germany).

## 3 Results

### 3.1 SNP identification

Utilising all 96 mRNA-Seq profiles for variant calling, we identified in total 4,482,290 SNPs. For a subset of 20 individuals, SNP data obtained using the Illumina porcine 60 K SNP array were available from other projects (Reyer et al., 2019). We analyzed correlation between genotype data collected by the two different methods for 2,468 shared SNPs. The individual correlations ranged between 0.936 and 0.955 demonstrating high reliability of genotypes called from the mRNA-Seq data. After removing outlier samples (as suggested by exploratory data analysis of gene expression/response to treatment described previously (Li et al., 2021)) and filtering, we retained mRNA-Seq profiles from 20 individuals (each included in all four conditions) comprising a set of 93,598 autosomal SNPs with adequate read depth of both alleles in at least one sample (Supplementary Table S1). These SNPs covered 7,321 non-overlapping genes, about a half of the genes detected by the mRNA-Seq experiment. Most of the SNPs in the final set (∼88%) are known variants listed in public databases.

It has to be noted that using our filtering criteria we essentially removed SNPs showing monoallelic expression. However, monoallelic expression is more relevant for the study of genomic imprinting rather than for the detection of *cis*-regulatory effects as aimed in this study. Yet, omission of this aspect enhanced reliability of the data due to the more stringent criteria, reducing the risk of miscalling homozygous SNPs.

Besides miscalled genotypes, allelic mapping bias is another major caveat in the ASE analysis (Castel et al., 2015). Therefore, we plotted distribution of the allelic ratios for the final SNP set to verify if the allelic mapping bias has been effectively removed (see the methods section). The allelic ratios (ref/ref + alt) showed approximately normal distribution (Supplementary Figure S1) and no apparent bias towards the reference allele (mean ratio 0.494), thus confirming successful correction.

### 3.2 Analysis of allele specific expression within condition

To identify informative loci for the G×E study, we first examined ASE separately within each condition. We performed analysis based on individual SNPs using the VADT tool (Tomlinson et al., 2021) and gene-wise analysis of ASE employing the GeneiASE software (Edsgärd et al., 2016), respectively.

The SNP-wise analysis revealed 16,015 significant ASE variants (FDR ≤0.05) representing 3,323 unique genes in the untreated samples (C), 15,958 SNPs (3,225 unique genes) in dexamethasone treated samples (D), 13,772 SNPs (2,949 unique genes) in LPS treated samples (L), and 13,845 SNPs (covering 2,888 unique genes) in the combined treatment (LD). Overall, 32,730 variants (covering 4,924 unique genes) showed significant ASE in at least one condition and at least one sample (Supplementary Table S2). Out of these, 4,429 SNPs (covering 981 unique genes) exhibited ASE in all treatments (Supplementary Figure S2). To assess to which extent the ASE variants correspond with known eQTLs, we compared a list of the identified ASE variants with blood (including macrophage) *cis*-eQTLs in the PigGTEx database (Teng et al., 2022). Out of the 32,730 identified ASE SNPs, 38.9% are associated with blood *cis*-eQTL in the PigGTEx database.

Before running GeneiASE, we removed SNPs that could not be assigned to a unique gene leaving 75,620 SNPs available for the analysis. A further restriction was that by default GeneiASE performs analysis for genes covered with at least two SNPs. Overall, GeneiASE examined ASE of 4,219 genes represented in at least one condition, whereby 1,184, 1,199, 1,068, and 1,072 genes exhibited significant ASE (FDR ≤0.05) in C, D, L, and LD, respectively (Supplementary Table S3). Out of these, 509 genes displayed significant ASE in all conditions (Supplementary Figure S3). When examining overlap between the two approaches, more than 95% genes detected by GeneiASE were also identified based on the analysis of individual SNPs using VADT (Supplementary Figure S4).

Taken together, the ASE analysis provided a novel list of genes influenced by *cis*-regulatory variation and the input for the subsequent examination of G×E.

### 3.3 Analysis of condition-dependent allele specific expression

To identify genes influenced by environmentally responsive *cis*-regulatory variation, we analyzed cd-ASE between the untreated control and each of the three treatments, respectively.

**FIGURE 1:**
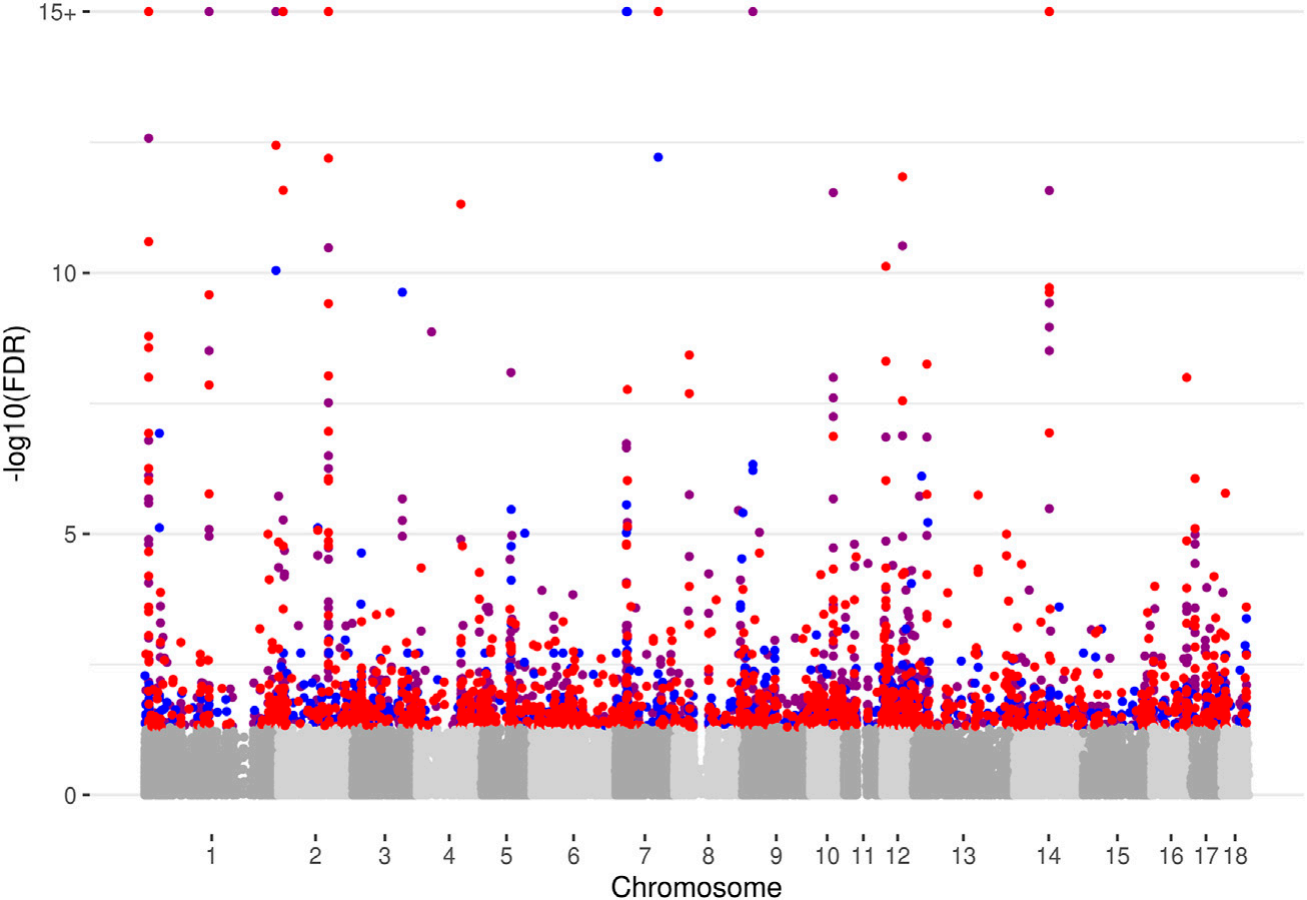
Manhattan plot depicting results of the analysis of conditional allele-specific expression. Significant cd-ASE loci (−log10 of Benjamini-Hochberg FDR >1.3) are shown in red for LPS compared to Control (C vs. L), in blue for Dexamethasone compared to Control (C vs. D), and purple for LPS + Dexamethasone compared to Control (C vs. LD), respectively. Non-significant loci are shown in grey tones. Please note that the −log10(FDR) values are flattened at −log10(FDR) = 15.

We examined individual SNPs using Fisher´s exact test, and restricted the analysis to those variants showing significant ASE in at least one sample and treatment (32,730 SNPs as described above). In addition, we included only variants passing the filtering criteria in both, the control as well as in the treatment condition for at least one sample. We identified significant cd-ASE (FDR ≤0.05) for 1,336 SNPs (covering 713 unique genes) in C vs. L comparison, for 904 SNPs (covering 574 unique genes) in C vs. D, and for 1,353 SNPs (covering 713 unique genes) in C vs. LD, respectively (Supplementary Table S4). An overview of the results is given in the manhattan plot in Figure 1. Only small fraction of the SNPs exhibiting cd-ASE came up in all three comparisons (Supplementary Figure S5). This could be expected, because the different treatments activate distinct *trans*-acting factors and thus distinct cistromes. When looking at SNPs that displayed significant cd-ASE in at least three samples, 558 SNPs came up in C vs. L, 374 SNPs in C vs. D, and 560 SNPs in C vs. LD, respectively.

Similar to the analysis of ASE within treatment, we complemented the investigation of individual cd-ASE with gene-wise analysis using GeneiASE. In keeping with the SNP-wise procedure, genes entering the gene-wise analysis were preselected, and only those showing static ASE in at least one sample and treatment were examined (2,604 genes in total). The gene-wise analysis yielded 15, 12, and 22 genes displaying significant cd-ASE (FDR ≤0.05) associated with L, D, and LD treatment, respectively (Supplementary Table S5). Most of the genes were detected also based on the analysis of cd-ASE of individual SNPs (Supplementary Figure S6).

Overall, the analysis of individual SNPs revealed more genes affected by cd-ASE compared to gene-wise analysis. Based on the SNP-wise analysis we found evidence for G×E for about ten percent of the ASE SNPs.

### 3.4 Investigation of LPS-dependent *cis*-regulatory effects on *SOD2* expression

**FIGURE 2:**
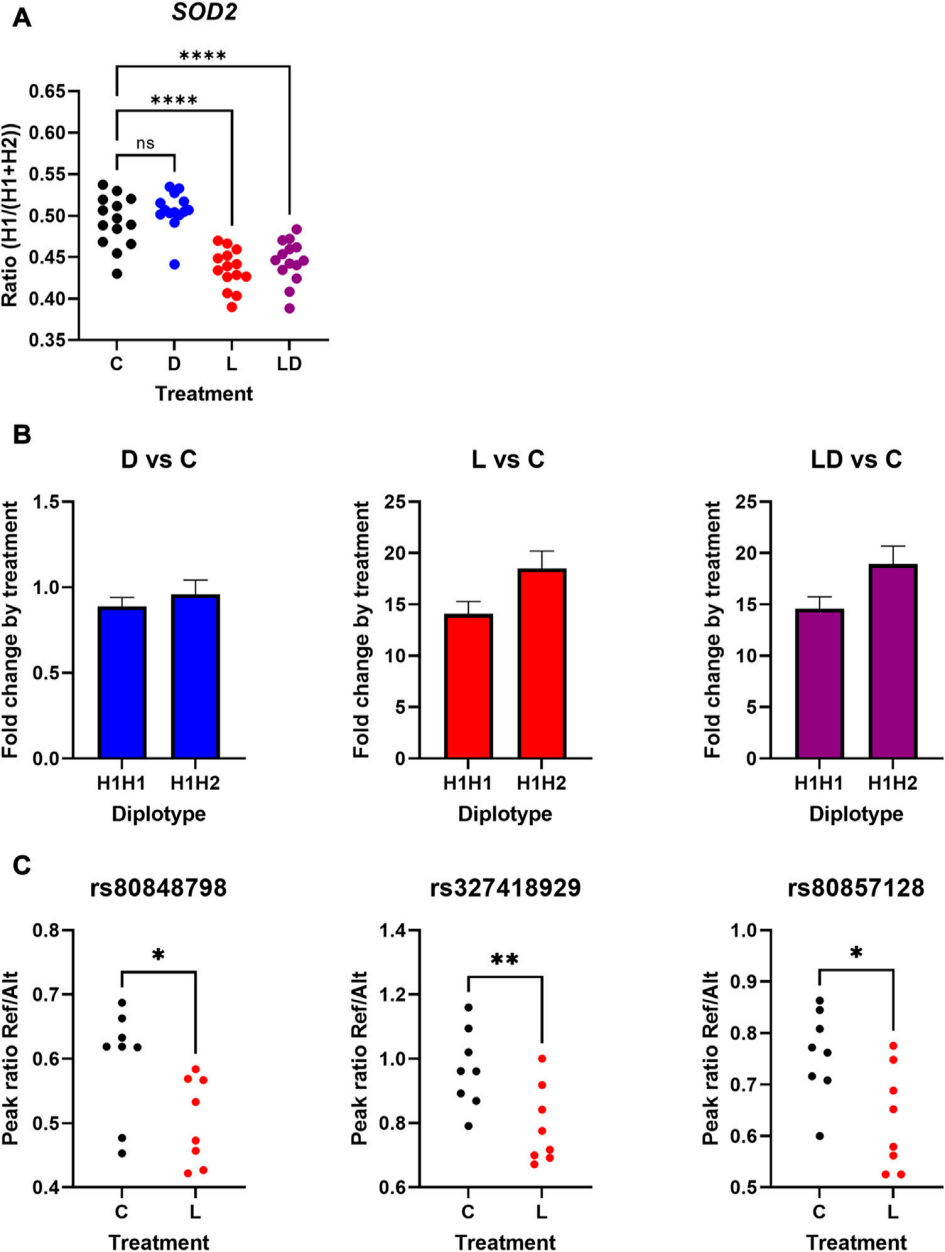
LPS-dependent *cis*-regulatory effects on the expression of the porcine *SOD2* in PBMCs. **(A)**: Haplotype-level allelic ratios of *SOD2* depending on treatment (*n* = 14 heterozygous samples). **(B)**: Fold-change response of *SOD2* to treatment depending on haplotype (H1H1 *n* = 6; H1H2 *n* = 14). **(C)**: Peak ratios of three *SOD2* SNPs depending on treatment of a different set of informative PBMCs (*n* = 8). Please note that the reference alleles were assigned to H1, and the alternative alleles to H2, respectively C: vehicle control. D: Dexamethasone treatment (blue). L: LPS treatment (red). LD: LPS + Dexamethasone treatment (purple). Asterisks indicate different significance level *p ≤ 0.05, **p ≤ 0.01, ***p ≤ 0.001, ****p ≤ 0.0001. ns = not significant.

One of the top candidates showing LPS-dependent ASE based on SNP-wise analysis is *SOD2*. This gene features one of the strongest transcriptional responses to LPS in monocytes (Li et al., 2021; Lara et al., 2022). *SOD2* encodes a mitochondrial antioxidant enzyme protecting cells from oxidative stress resulting i.a. from the inflammatory response induced by LPS, and thus supporting its resolution (Virág et al., 2019). Out of the 21 informative cSNPs interrogating *SOD2*, 15 SNPs showed significant cd-ASE associated with the LPS treatment. In spite of this, *SOD2* was not detected by GeneiASE as a gene influenced by cd-ASE. To verify cd-ASE of *SOD2*, we first analyzed haplotype structure and haplotype-level cdASE of *SOD2*. We found two haplotypes (designated H1 and H2, respectively) segregating in the 20 examined individuals (i.e., those used to produce the mRNA-seq data)—six being homozygous for the wild-type haplotype H1 and fourteen heterozygous (Supplementary Figure S7). We calculated haplotype-level allelic ratio for the 14 informative heterozygous samples and compared the ratios between treatments. As displayed in Figure 2A, the haplotype ratios measured in C (mean 0.493) and D (mean 0.506) were not significantly different and were close to balanced expression. In contrast, in L and LD treatments the haplotype ratios shifted significantly compared to C (mean 0.435 and 0.445, respectively), indicating higher responsiveness of the alternative *SOD2* haplotype H2 following exposure of the PBMC to LPS.

To further examine the influence of *SOD2* haplotypes on its transcriptional response to treatment, we analyzed association of the diplotype with the fold-change between treatment and control conditions based on normalized total count data retrieved from the previous study (Li et al., 2021). As depicted in Figure 2B, the heterozygous carriers showed stronger response to LPS, and LPS in the presence of DEX, respectively, compared to homozygous carriers. The direction and magnitude of the effect of the H2 haplotype corresponds with the haplotype ratio calculated in the above analysis, but the H2 effect was not significant here.

In order to independently verify the LPS-dependent ASE of *SOD2*, we performed LPS-stimulation for a different set of informative PBMC samples, and analyzed cd-ASE for three of the significant cSNPs in eight heterozygous samples *via* a targeted assay bases on Sanger sequencing. As outlined in Figure 2C, for all three cSNPs the LPS treatment significantly (*p* < 0.05) shifted the allelic ratio towards the alternative allele (assigned to haplotype H2).

Collectively, these results provide strong evidence for LPS-dependent *cis*-regulatory effects on *SOD2* expression.

### 3.5 Functional enrichment of genes exhibiting cd-ASE

**FIGURE 3:**
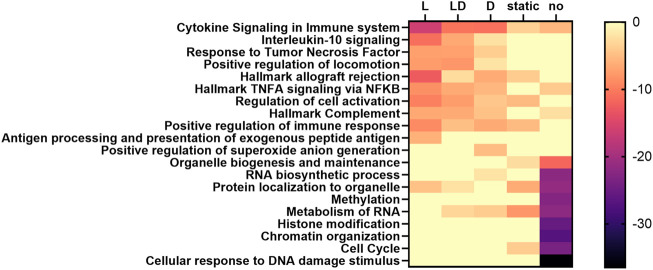
Heatmap of the enrichment (log10-transformed *p*-values) of selected functional terms among genes with different ASE characteristics, predicted by Metascape. L: set of genes showing cd-ASE depending on LPS. D: set of genes showing cd-ASE depending on Dexamethasone. LD: set of genes showing cd-ASE depending on LPS + Dexamethasone. Static: set of genes exhibiting ASE across treatments but no cd-ASE. No: set of genes showing no significant ASE.

In order to identify potential regulatory mechanisms driving cd-ASE, we functionally annotated genes with at least three significant cd-ASE samples in the SNP-wise analysis (299 genes in C vs. L, 259 genes in C vs. D, 302 genes in C vs. LD) compared to genes exhibiting exclusively static ASE across treatments (i.e., ASE but no cd-ASE) in at least three samples (489 genes), and genes showing no evidence of ASE (2,397 genes). Pathway analysis revealed enrichment of genes showing no or static ASE in functional categories related to cell cycle, cellular response to DNA damage, and chromatin organization (Supplementary Table S6; Figure 3). Correspondingly, transcription factors involved in cell cycle regulation (e.g., CCND1 for no ASE genes) and proliferation (e.g., MYC) emerged when upstream regulators were examined for the genes that showed no evidence of cd-ASE (Supplementary Table S7). These results suggest selection against variation in genes related to cell proliferation, which could be a mechanism protecting against cancer (Hanahan and Weinberg, 2011).

Genes influenced by cd-ASE were enriched i.a. for cytokine signaling, particularly for interleukin 10 and tumor necrosis factor response in treatments including LPS (Supplementary Table S6; Figure 3). The top upstream regulators included canonical members of the LPS signaling pathway such as MYD88 and NFKB in genes with cd-ASE induced by the LPS treatment (Supplementary Table S7). For genes with dexamethasone induced cd-ASE the predicted upstream regulators included, for example, the transcription factor GATA3, a known target of the glucocorticoid receptor (Liberman et al., 2009). The combined treatment LD shared most of the top upstream regulators with the specific treatments.

## 4 Discussion

In the present study, we performed the first comprehensive examination of G×E effects on gene expression in farm animals using the analysis of condition-dependent allele-specific expression. We investigated cd-ASE in response to stimuli that were also frequently used in human studies (e.g., Moyerbrailean et al., 2016; Knowles et al., 2017). The applied treatments do not only model relevant responses to common challenges in farm animals, but also induce vast gene expression changes *via* well-described signaling pathways (Li et al., 2021) and are thus ideally suited to study cd-ASE. Moreover, the application of these agents to PBMC *in vitro* provided standardized conditions, and in line with the 3R principles in animal experiments avoided animal stress. In fact, *in vitro* models open up new possibilities in the analysis of G×E interactions for environmental factors that are difficult to examine on live animals. In addition, using cell models facilitates biologically better-founded interpretation of the results. In this regard, future application of cd-ASE analysis on single-cell RNA-sequencing profiles would bring further improvement because it is likely that many G×E interactions occur in cell-type specific manner (Findley et al., 2021).

We first performed ASE analysis for each condition separately to identify informative loci for the cd-ASE analysis, and thus to reduce the multiple testing burden for the latter. Out of the 32,730 identified ASE SNPs more than 60% do not overlap with *cis*-eQTL SNPs in the recently released PigGTEx database (Teng et al., 2022). In addition, less than 15% of the significant ASE variants showed ASE in all treatments. These findings imply that ASE analysis across different environmental conditions and/or cell types has the potential to uncover novel *cis*-regulatory effects, as previously observed in ASE studies in humans (Findley et al., 2021). On the other hand, about two thirds of all genes interrogated by the tested cSNPs showed ASE in at least one treatment and sample. This is consistent with the high prevalence of *cis*-regulatory variation affecting majority (around 90%) of all protein-coding genes, with more than 20% showing evidence for multiple independent *cis*-regulatory variants (GTEx Consortium, 2020; Teng et al., 2022).

For the analysis of cd-ASE we performed SNP-wise as well as gene-wise analysis according to Edsgärd et al. (2016). A current limitation in the analysis of cd-ASE is that the approach has not yet found broad application, and consequently the tools and pipelines are not yet firmly established. The advantage of the approach used here is that, in contrast to many other tools for cd-ASE analysis (e.g., EAGLE, ASEP), here not only loci but also samples showing significant cd-ASE were identified, which may support future identification of the causal *cis*-regulatory variants. In addition, the information about the number of informative samples exhibiting cd-ASE allows conclusions about the strength of the evidence. In our study analysis of individual SNPs detected more loci exhibiting cd-ASE compared to the gene-wise GeneiASE method, as also noted previously by Salavati et al. (2019), but with a high degree of overlap between the two methods. We independently confirmed cd-ASE of *SOD2*, one of the top candidates based on the SNP-wise analysis, which was missed by the gene-wise analysis. This result corroborates the advantage of the SNP-wise analysis over the gene-wise approach implemented by the GeneiASE method.

In order to verify LPS-dependent *cis*-regulatory effects on *SOD2* we used different methodologies including haplotype-level ASE analysis, and analysis of LPS-response depending on the diplotype. Although both methods pointed to enhanced LPS-responsiveness of the H2 haplotype of *SOD2*, only results from the haplotype-based ASE analysis were significant, demonstrating higher power of the ASE approach compared to the reQTL analysis, particularly for traits such as the LPS-response with inherently high individual variation. In fact, we were able to confirm the enhanced transcriptional responsiveness of the H2 haplotype in an independent cell experiment coupled with locus-specific ASE analysis. Further research is needed to identify the underlying *cis*-regulatory variation. Notably, the PigGTEx database contains *SOD2* eQTLs in nine different tissues. The most significant SNPs influence *SOD2* expression in the blood, and reside at the 3′ end of the gene, suggesting the most likely location of the causal variation. Considering the important function of *SOD2* in the innate immunity (Peterman et al., 2015) and generally beneficial effects of superoxide dismutases during inflammation (Carillon et al., 2013; Ahasan et al., 2019), it could be speculated that the H2 haplotype, or more precisely, the causal variation, is likely associated with enhanced disease resistance. This hypothesis needs further investigation, which may be facilitated by the presence of one of the validated cd-ASE SNPs, rs80857128, on the Illumina porcine SNP array.

The list of genes where both methods evidenced cd-ASE includes highly relevant candidates such as *IDO1*. The gene is strongly activated in response to inflammatory stimuli, including LPS (Li et al., 2021). Accordingly, *IDO1* showed cd-ASE associated with LPS application (i.e., in C vs. L as well as C vs. LD). It encodes an enzyme that catalyzes the initial rate-limiting step in the conversion of tryptophan *via* the kynurenine pathway. The resulting depletion of tryptophan and bioactive metabolites produced by *IDO1* have an immunomodulary function (Pallotta et al., 2022). Importantly, these metabolites also possess neuromodulatory activity, linking the immune response and *IDO1* to (damaging) behaviour (Nordgreen et al., 2020).

Regarding the DEX treatment, the strongest evidence for cd-ASE was found for *OLR1*, which was detected by both SNP- and gene-wise analysis. The *OLR1* gene encodes a receptor (LOX1) for oxidatively modified low density lipoprotein (oxLDL), a key proatherogenic factor. Uptake of oxLDL by monocytes promotes their aggregation and adhesion to vascular endothelial cells, differentiation into foam cells and atherosclerosis (Frostegård et al., 1990). Interestingly, *OLR1* exhibited also LPS-dependent ASE, but only in the SNP-wise analysis. The *OLR1* gene is upregulated by LPS (Li et al., 2021), and other pro-inflammatory stimuli, connecting inflammation to atherogenesis (Pirillo et al., 2013). In contrast to LPS and DEX treatments, we found no evidence for cd-ASE of *OLR1* in the combined LD treatment. Taken together, these findings suggest that glucocorticoids (such as DEX) most likely modify the risk of atherosclerosis conferred by the *cis*-regulatory variation of *OLR1*.

## 5 Conclusion

The present study demonstrates the utility of functional genomics for the investigation of G×E. We applied different methods of cd-ASE analysis and as evidenced by the example of *SOD2* cd-ASE, found that SNP-wise analysis yields more meaningful results. Future studies would benefit from methodological developments such as haplotype- or single cell RNA sequencing-based cd-ASE analysis. We found strong evidence for environmentally sensitive *cis*-regulatory variation in several prominent candidates for pig health and welfare. Overall, the genes affected by cd-ASE are enriched in cytokine signaling pathways. Thus, the established collection of SNPs and genes showing ASE and cd-ASE may aid future efforts to identify loci influencing immune response, and may even be directly considered in genetic improvement of pig health by the currently evolving methods to incorporate functional annotation into genomic prediction models (Shi et al., 2022).

## Data Availability

The datasets presented in this study can be found in online repositories. The names of the repository/repositories and accession number(s) can be found below: https://www.ebi.ac.uk/arrayexpress/, E-MTAB-9808.
