# Supplementary material for: Exploration of genotype-by-environment interactions affecting gene expression responses in porcine immune cells
Source: Front Genet. 2023 Mar 16;14:1157267. doi: 10.3389/fgene.2023.1157267 (PMC10061014; doi:10.3389/fgene.2023.1157267)
Supplement: Supplementary file 8 [file Image1.pdf]

*Supplementary Material*

# Exploration of genotype-by-environment interactions affecting gene expression responses in porcine immune cells

**Eduard Murani*, Frieder Hadlich**

*** Correspondence:** Eduard Murani: murani@fbn-dummerstorf.de

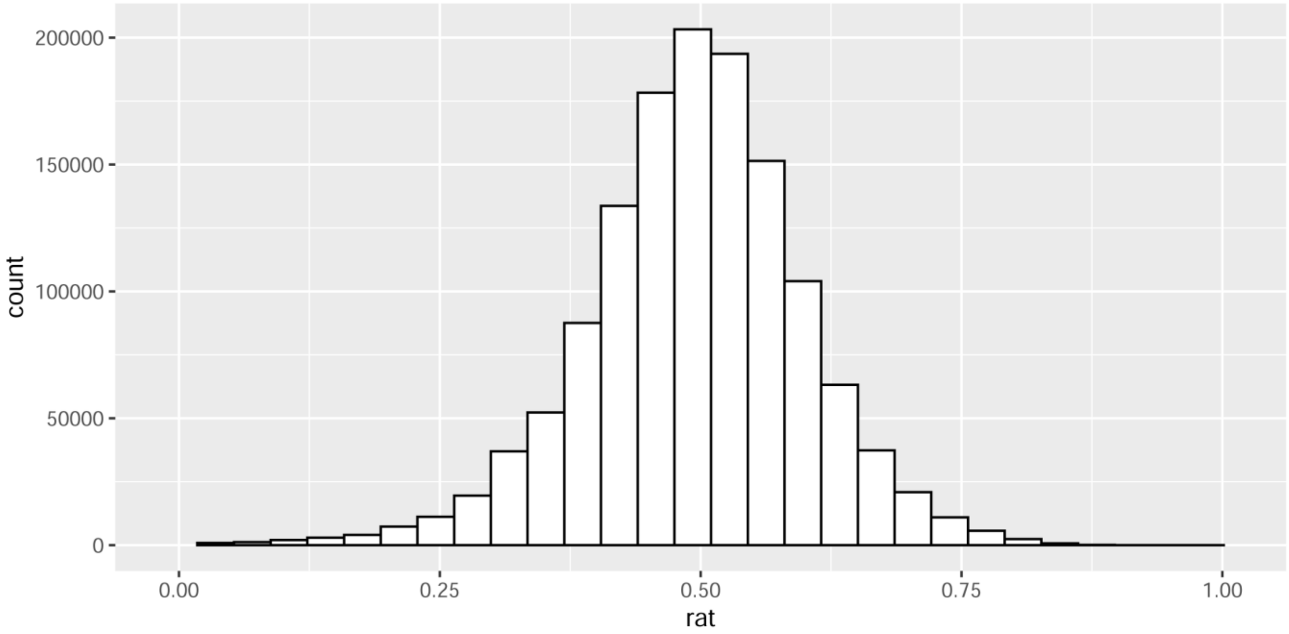


**Supplementary Figure 1.** Histogram of allelic ratios (x-axis, ref/ref+alt) of the 93598 autosomal SNPs used for the analysis of allele-specific expression.

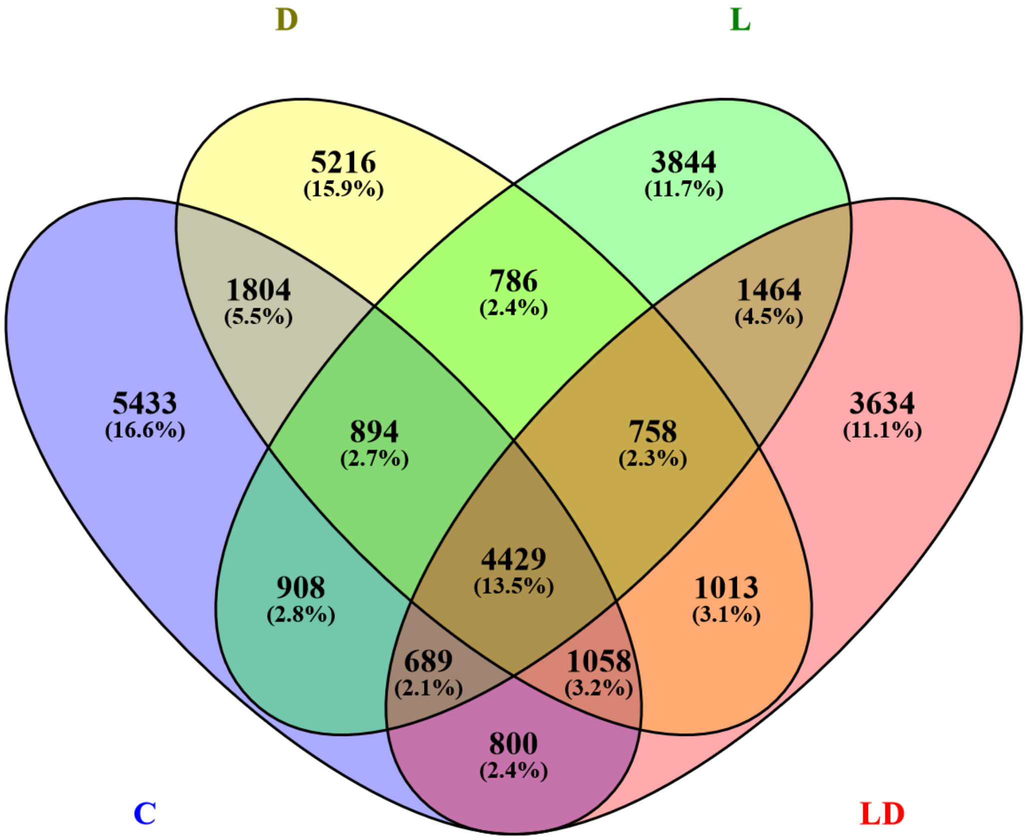


**Supplementary Figure 2.** Overview of the number of SNPs showing allele-specific expression within each treatment of porcine PBMC *in vitro*. C: vehicle. D: Dexamethasone. L: LPS. LD: LPS+Dexamethasone.

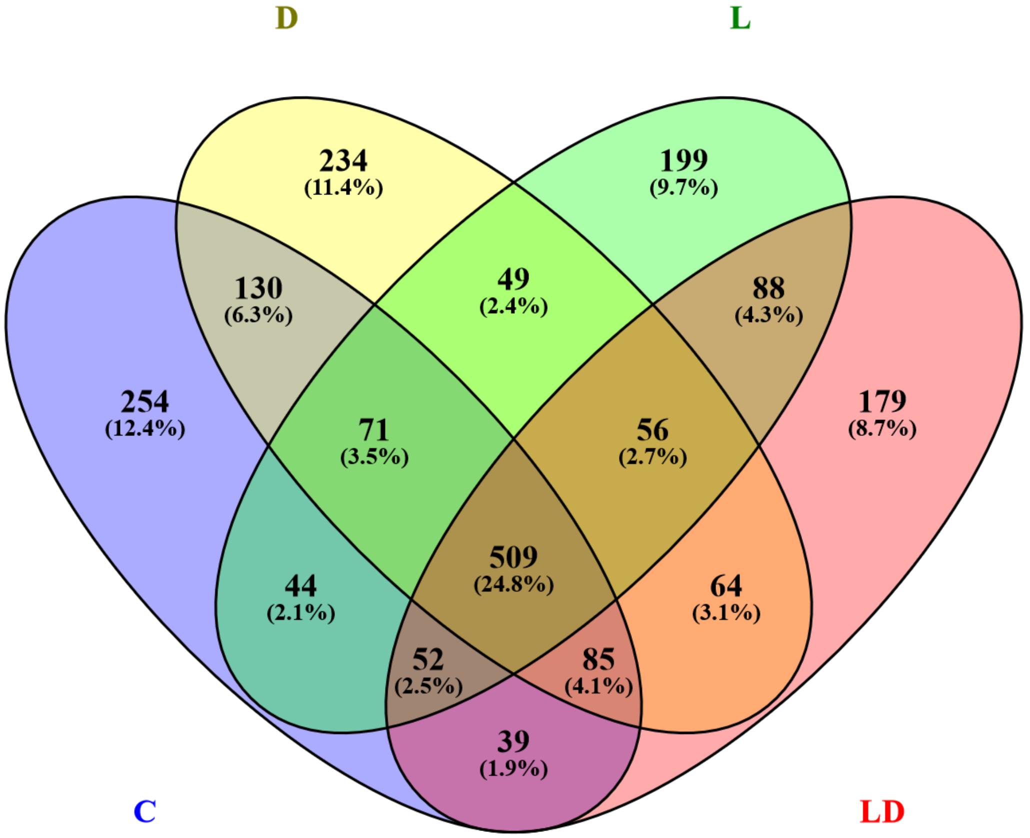


**Supplementary Figure 3.** Overview of the number of genes showing allele-specific expression in GeneiASE analysis within each treatment of porcine PBMC *in vitro*. C: vehicle. D: Dexamethasone. L: LPS. LD: LPS+Dexamethasone.

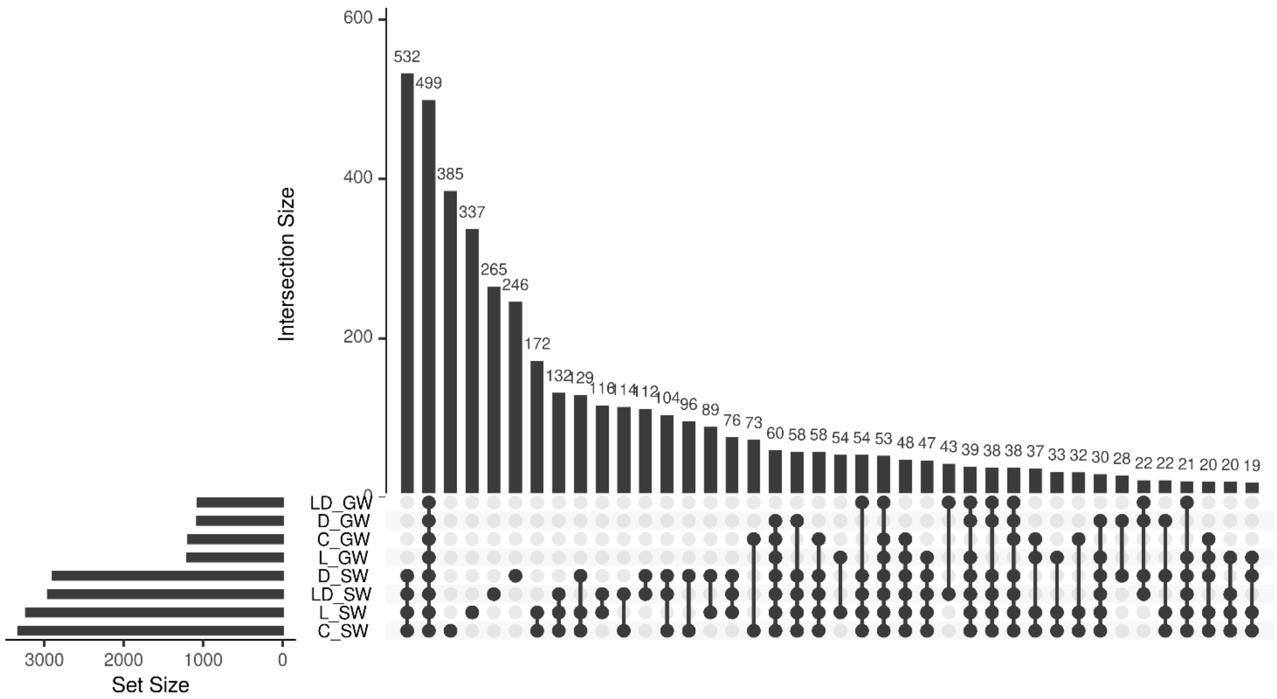


**Supplementary Figure 4.** Overview of the overlap between genes detected by SNP-wise and gene-wise analysis of allele-specific expression in porcine PBMC in vitro. C: vehicle. D: Dexamethasone. L: LPS. LD: LPS+Dexamethasone. SW: SNP-wise analysis. GW: Gene-wise analysis

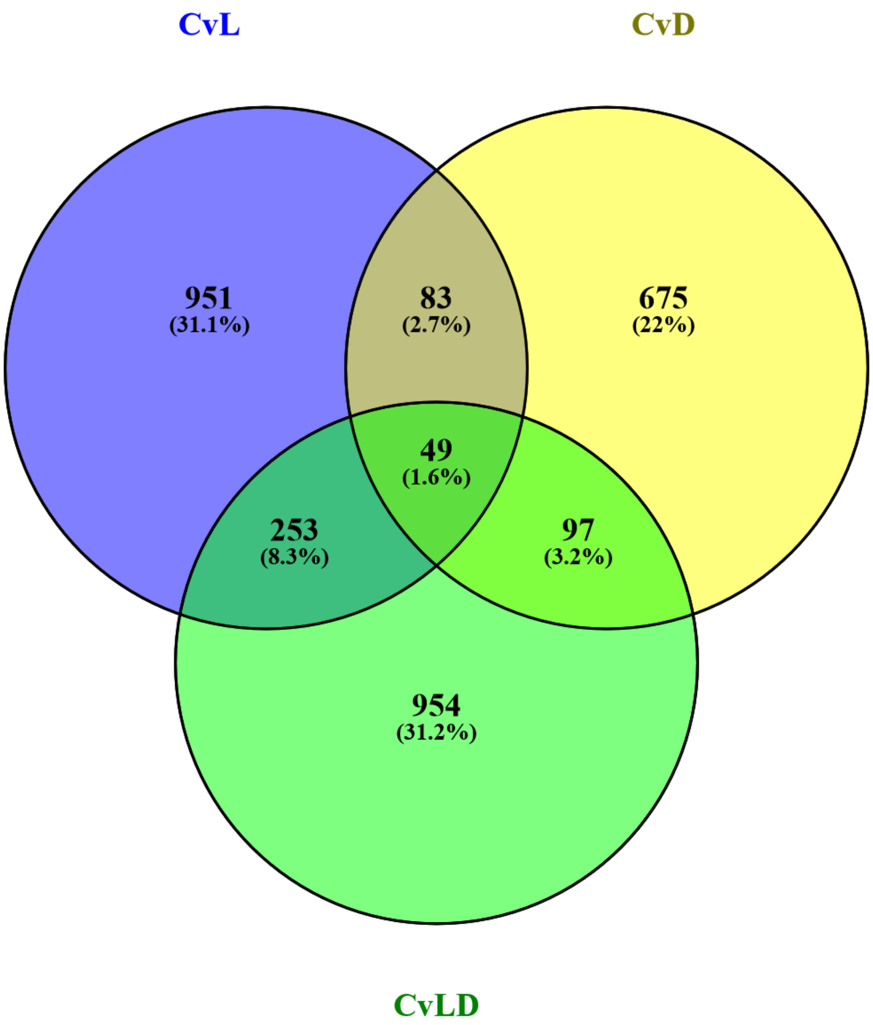


**Supplementary Figure 5.** Overview of the number of SNPs showing condition-dependent allele-specific expression in porcine PBMC *in vitro*. C: vehicle. D: Dexamethasone. L: LPS. LD: LPS+Dexamethasone.

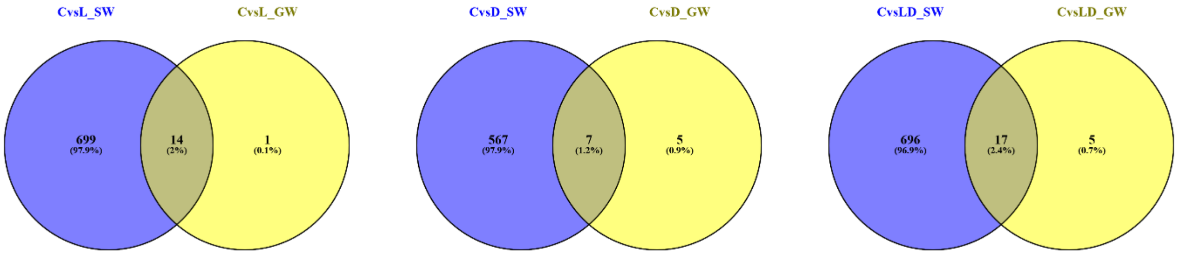


**Supplementary Figure 6.** Venn diagrams of the overlap between genes detected by SNP-wise and gene-wise analysis of condition-dependent allele-specific expression in porcine PBMC *in vitro*. C: vehicle. D: Dexamethasone. L: LPS. LD: LPS+Dexamethasone. SW: SNP-wise analysis. GW: Gene-wise analysis

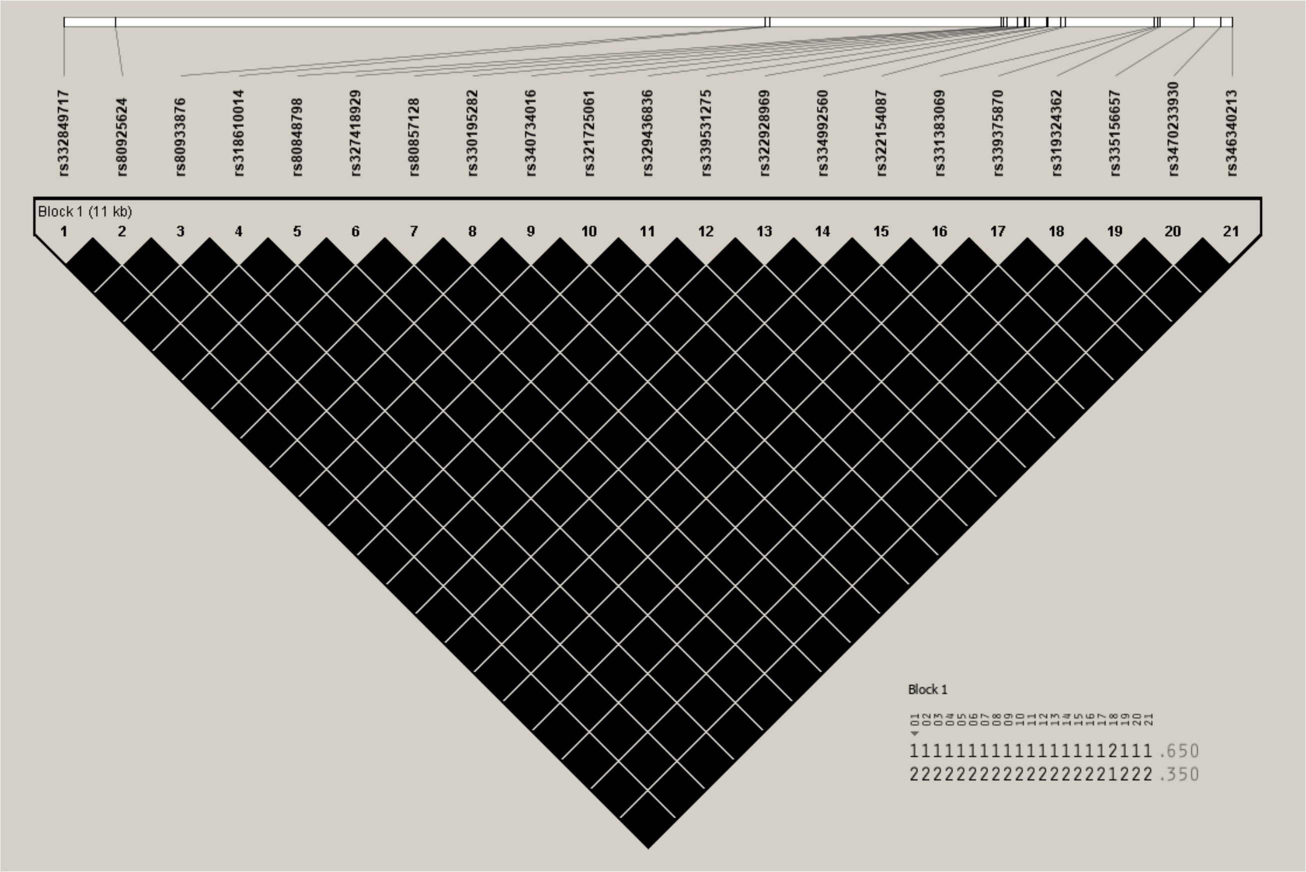


**Supplementary Figure 7.** Haplotype structure of the porcine *SOD2* in the analyzed PBMC samples. This was derived from genotype data of 21 SNPs called from mRNA-Seq data and used in the analysis of allele-specific expression. Haplotype block was defined using the four gametes rule. Black squares indicate r squared equal to 1.
